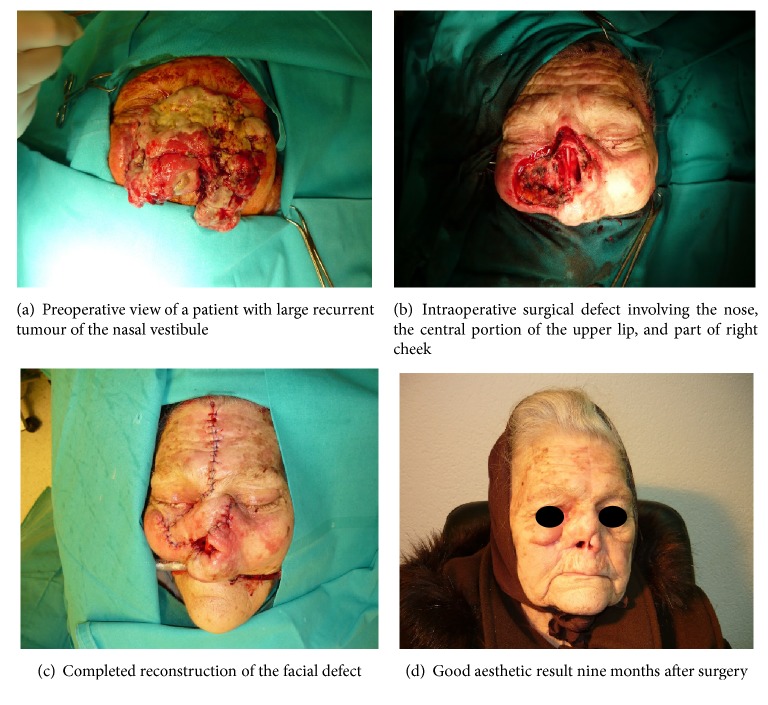# A Rare Case of Recurrent Mucoepidermoid Carcinoma of the Nasal Vestibule

**DOI:** 10.1155/2017/1421204

**Published:** 2017-09-13

**Authors:** Vladimir Bedeković, Miro Leventić, Boris Jelavić, Robert Trotić, Mihael Ries, Mirjana Kostić, Tomislav Lauc

**Affiliations:** ^1^School of Medicine, Department of Otorhinolaryngology and Head and Neck Surgery, Sisters of Mercy University Hospital Center, University of Zagreb, Zagreb, Croatia; ^2^School of Medicine, Department of Otorhinolaryngology and Head and Neck Surgery, University of Mostar, Mostar, Bosnia and Herzegovina; ^3^School of Medicine, Croatian Health Insurance Fund, Zagreb, Croatia; ^4^School of Dental Medicine, Dental Clinic Apolonia, Zagreb, Croatia

## Abstract

We report a rare case of a large recurrent mucoepidermoid carcinoma (RMEC) in an 81-year-old female smoker, which has originated in the right nasal vestibule. The recurrent tumour was inadequately treated for 6 years. It was a slow-growing tumour for 3 years and then began to enlarge at a higher pace. In the next three years it has covered a large part of the face. The patient had refused any medical treatment. The tumour caused breathing and swallowing difficulties. Because of the profuse bleeding from the tumour, the patient underwent emergency surgery. Surgical treatment consisted of rhinectomy and resection of the central upper lip and part of the right cheek. The facial defect was reconstructed immediately. Recovery from surgery was fast with no complications. Postoperative Multislice Computed Tomography scan showed no metastases so the patient did not receive any chemotherapy or radiotherapy. During a 2.5 years' follow-up period there was no recurrence of the disease.

## 1. Introduction

Mucoepidermoid carcinomas (MEC) of the nasal vestibule (NV) are extremely rare. Only a few cases were reported. MEC is the most common primary carcinoma of major and minor salivary glands of the oral cavity and pharynx, the lacrimal glands, and the parotid. It accounts for less than 1% of all malignancies of the head and neck.

The pathogenesis is not known. [1–9]. The guidelines for cancers in the nasal area are not uniformly accepted. Recurrent carcinomas of the nasal vestibule have never been studied as a specific entity. The prognosis of MEC depends on many factors including early diagnosis, histological grade, and the clinical stage of the tumour. Treatment options include surgery, radiotherapy, and chemotherapy [10–12]. MEC of the NV is usually a low-grade and slow-growing cancer with very rare metastases which are often treated only with surgical resection, depending on the extent of the disease. Combined surgery and postoperative radiotherapy has been advocated for intermediate and high-grade tumours [13, 14]. Some studies have found that smoking tobacco, alcohol consumption, and previous radiation exposure might increase the risk of NV cancer [15].

## 2. Case Report 

An 81-year-old female smoker with cardiac arrhythmia is presented with a 6-year history of inadequate treatment of recurrent mucoepidermoid carcinoma (RMEC) originating from the right NV. Nine years ago, the patient had experienced breathing difficulties through her right nostril for 3 weeks. Nasal endoscopy revealed a 1 × 1 cm, painless, fixed, round, smooth-surfaced, protruding mass in the right nasal vestibule. Neck examination showed no enlarged lymph nodes. The tumour mass was fully excised and histologically identified as low-grade mucoepidermoid carcinoma. The patient did not receive any therapy. The tumour has recurred within 6 months. A biopsy confirmed the previous diagnosis. Multislice Computed Tomography (MSCT) scan showed no regional or distant metastatic spread, and the patient has refused surgical or any other medical treatment. RMEC was a slow-growing tumour for 3 years and then began to enlarge at higher pace. In the next three years, it has affected almost the whole face: the nasal septum, columella, philtrum, and a large part of both cheeks. The tumour bled occasionally. The patient's condition has deteriorated in time. In spite of her very serious condition, the patient once again refused medical treatment. On the day of admission to the hospital the tumour bled profusely, and the patient underwent emergency surgery. There was no time for radiological examination (Figure 1(a)). The patient received the blood transfusion before, during, and after surgery. Surgical treatment consisted of rhinectomy and resection of the central upper lip and part of the right cheek (Figure 1(b)). The large facial defect was reconstructed immediately with the vertical paramedian forehead flap, cheek flap, and nasolabial flap (Figure 1(c)). Histology finding confirmed a low-grade MEC. Seven days after surgery the patient's general condition was good; respiration, speech, and eating were normal. Senses of taste and smell were well-preserved.

## 3. Discussion 

We found no articles addressing the recurrent mucoepidermoid carcinoma of the nasal vestibule in available literature. As of our knowledge, this is the first described case. In our patient a large RMEC caused breathing, speaking, chewing, swallowing, eating, and sleeping difficulties. Impaired sense of smell, facial pain, and headache were not present. The bad body odor and fetid nasal discharge were emitted as a result of accompanying paranasal sinusitis, and as a result of malacia of decomposed tumour masses. RMEC was histopathologically diagnosed as a low-grade malignant tumour following biopsy, which was confirmed after tumour excision. Postoperative MSCT scan showed no regional or distant lymph node metastases, so there was no need for chemotherapy or radiotherapy. Cardiac arrhythmia and secondary anemia were well-controlled by adequate therapy. Nine months after the operation the aesthetic aspect was more than satisfactory (Figure 1(d)). No recurrence of the disease was noted during a follow-up period of 2.5 years.

## 4. Conclusion

In case of a large, profusely bleeding tumour surgery is a procedure of choice.

For treatment of recurrent mucoepidermoid carcinoma delayed surgery may be successful.

Mucoepidermoid carcinoma of the nasal vestibule is extremely rare, and it has never been studied as a specific entity. Treatment of mucoepidermoid carcinomas has no uniformly accepted guidelines, and each case has to be treated individually.

## Figures and Tables

**Figure 1:**